# Penalized-regression-based multimarker genotype analysis of Genetic Analysis Workshop 17 data

**DOI:** 10.1186/1753-6561-5-S9-S92

**Published:** 2011-11-29

**Authors:** Kristin L Ayers, Chrysovalanto Mamasoula, Heather J Cordell

**Affiliations:** 1 Institute of Genetic Medicine, Newcastle University, International Centre for Life, Central Parkway, Newcastle upon Tyne, NE1 3BZ, UK

## Abstract

Testing for association between multiple markers and a phenotype can not only capture untyped causal variants in weak linkage disequilibrium with nearby typed markers but also identify the effect of a combination of markers. We propose a sliding window approach that uses multimarker genotypes as variables in a penalized regression. We investigate a penalty with three separate components: (1) a group least absolute shrinkage and selection operator (LASSO) that selects multimarker genotypes in a gene to be included in or excluded from the model, (2) an allele-sharing penalty that encourages multimarker genotypes with similar alleles to have similar coefficients, and (3) a penalty that shrinks the size of coefficients while performing model selection. The penalized likelihood is minimized with a cyclic coordinate descent algorithm, allowing quick coefficient estimation for a large number of markers. We compare our method to single-marker analysis and a gene-based sparse group LASSO on the Genetic Analysis Workshop 17 data for quantitative trait Q2. We found that all of the methods were underpowered to detect the simulated rare causal variants at the low false-positive rates desired in association studies. However, the sparse group LASSO on multi-marker genotypes seems to provide some advantage over the sparse group LASSO applied to single SNPs within genes, giving further evidence that there may be an advantage to modeling combinations of rare variant alleles over modeling them individually.

## Background

It has previously been shown that multi-locus data analysis can improve power to detect causal variants [1]. Regression methods offer an attractive alternative to single-marker testing in genetic association analysis, and allow us to model the effect of several genes or several markers within a gene simultaneously. In particular, penalized regression methods are useful in underdetermined problems where the number of predictors is larger than the number of observations, and have been shown to improve power over single-locus test when there are multiple causal variants [2]. Penalized regression methods shrink down to zero the coefficient of markers that have little apparent effect on the trait of interest, resulting in a parsimonious subset of what we hope are true predictors. See Dasgupta et al. ([3], sec. 3.1) for background information on penalized/regularized regression methods.

As an alternative to modeling SNP markers as predictors in regression, we can use haplotypes or multi-marker genotypes as predictors. These are useful not only for capturing untyped variants but also for looking at the effects of combinations of alleles in close proximity. We can use a group penalized regression method to encourage variables within a region (such as SNPs in the same gene or multi-marker genotypes spanning the same markers) to enter the model as a group. With multi-marker genotypes (or alternatively haplotypes), an allele-sharing penalty can be used to encourage multi-marker genotypes that share rare alleles to have similar coefficients.

The Genetic Analysis Workshop 17 (GAW17) mini-exome data is based on sequence data in which many rare variants are included. SNPs were genotyped within 3,205 genes providing a natural grouping for testing the methods mentioned above. We used the quantitative trait Q2 to compare methods using both single-markers and multiple-markers as predictors of Q2. For the single-locus association tests, all 24,487 SNPs were used individually. For the grouping methods, the 24,487 SNPs were divided into 3,205 gene groups for each analysis.

## Methods

### Analytical approach

Quantitative traits can be analyzed by minimizing the sum of square residuals (RSS). Given a phenotype vector *Y* of *m* observations and a matrix of *p* multimarker single-nucleotide polymorphism (SNP) genotypes *X*, we estimate our vector of regression coefficients *β* by minimizing:(1)

The multimarker genotypes for a set of SNPs are defined as the combination of genotypes at the set of SNPs in question, where the genotype at an individual SNP can be collapsed a priori if required (e.g., using dominant or recessive coding). For example, a set of three SNPs would generate 2^3^ possible multimarker genotypes if they were collapsed using binary coding. Each multimarker genotype is considered a predictor variable in Eq. (1), where *x_ij_* = 1 for individual *i* if that individual has multimarker genotype *j* and *x_ij_* = 0 otherwise.

### Penalization

Penalized regression methods constrain the size of the regression coefficients and are used mainly for two purposes: (1) variable selection and (2) controlling the size of estimated coefficients for rare variables that often have high variances. Penalized regression methods permit the use of regression in underdetermined problems, where the number of variables is far larger than the number of observations. For a quantitative trait, our objective function to be minimized can be written:(2)

where the penalty *f* is a function of the regression coefficients (and possibly a mixing parameter). The rate of shrinkage is directly controlled by the derivative of the penalty function, and many different penalty functions have been proposed.

In our method, each multimarker genotype is considered a predictor variable. This uncoupling removes any dependence between the multimarker genotypes, even if they share the same markers and are thus not independent. To this end, we would like to encourage SNPs within a gene (in the form of multimarker genotypes) to be in the model together. We propose a three-part penalty that (1) encourages multimarker genotypes within a gene to be included or excluded as a whole (group penalty), (2) forces similar multimarker genotypes in a SNP window to have similar coefficients (an allele-sharing penalty), and (3) encourages overall sparsity while reducing the relative bias on large coefficients (minimax concave penalty [MCP]).

If *g* is a gene window, *G* is the total number of gene windows, and *j* indexes multimarker genotypes, we can write our penalty function as:(3)

where *λ* is the overall penalty strength, *θ* = (*θ*_1_, *θ*_2_, *θ*_3_) determines the strength of each penalty relative to the others, *L_g_* is the total number of multimarker genotypes in the gene, *w_j_* = (*h_j_*)^1/3^ is a weight based on the multimarker genotype frequency *h_j_*, and:(4)

where *a* is a tuning parameter that affects the range over which the penalty is applied. For a dominant/recessive genotype coding scheme, we define *ρ_ij_*, the allele-sharing statistic between two multimarker genotypes in the same marker window, as:(5)

where *A_ij_* is the *kth* allele of multimarker genotype *i*, *p_k_* is the allele frequency of the matching allele at marker *k*, and *K* is the number of markers in the window.

The idea is to penalize the difference in coefficients between two multimarker genotypes more heavily when they share at least one minor allele at a locus. For example, if two multimarker genotypes are homozygous for the common allele at a particular locus, we would slightly suspect that both of these multimarker genotypes would have an effect (if any) in the same direction, and thus we would add a small penalty if the coefficients of these two multimarker genotypes were quite different. Likewise, if both multimarker genotypes shared at least one minor allele, we would strongly suspect that the effect of possessing that allele (if any) was in the same direction and would thus incorporate a slightly stronger penalty. If one multimarker genotype in a pair is homozygous for the common allele at a locus and the other multi-marker genotype has at least one minor allele, then this position is not informative for this multimarker genotype pair, and thus we do not add any additional penalty. The allele-sharing penalty may be somewhat oversimplified for genotypes, but it provides us with a penalty that meets our design requirements and can be used as is with haplotypes. If a gene is large, we might break it up into smaller windows to limit diversity, and therefore a window may or may not cover a whole gene. Although a pair of multimarker genotypes may be in the same group or gene, their allele-sharing statistic will always be 0 when they do not share the same markers.

The first part of the penalty in Eq. (3), the group least absolute shrinkage and selection operator (LASSO) [4], encourages sparsity of groups (genes). The group LASSO penalty, does not force the coefficients to be equal but penalizes coefficients in a group less than coefficients in separate groups; that is,(6)

For instance, if other variables in the same group are already in the model, then a variable will receive a stronger push to enter the model than it would if, alternatively, it was in a group by itself. Zhou et al. [5] previously applied the sparse group LASSO to SNPs grouped within genes.

The allele-sharing penalty, the second part in Eq. (3), encourages coefficient estimates to move toward each other (so the difference between them is reduced) when there is high sharing between a pair of multimarker genotypes. This idea has previously been applied to haplotypes. Tzeng and Bondell [6] used a smoothing function, an L1 penalty term on pairwise differences,(7)

to force the estimates of haplotypes with the same effects to be exactly equal. Instead of variable selection or inference stabilization, Tzeng and Bondell’s goal was to identify haplotypes with the same effects by using an adaptive weight *w_h_*_,_*_h′_* related to the haplotype counts and the previous difference between the estimates. Haplotypes were collapsed into a group structure instead of looking at relative effects compared with a baseline haplotype, as normally done in regression with haplotypes.

We do not necessarily want to force the coefficients to be equal, but we do want them to be similar, for instance, to have the same sign. Our penalty is akin to the similarity penalty used by Tanck et al. [7]. Their allele-sharing statistic, expressed as the number of alleles that a pair of haplotypes share, results in the coefficients corresponding to rare haplotypes being smoothed toward the coefficients of a similar common haplotype. However, our sharing statistic depends on the frequency of the alleles shared. We define our sharing statistic in this manner because it is more likely that multimarker genotypes sharing rare alleles will either have a more recent ancestral haplotype or share spontaneous mutations. In either case, they should have a more similar effect than those that share only common alleles. In the case of spontaneous mutations, we are assuming that rare mutations, if causal, have a stronger effect. Thus we are encouraging multimarker genotypes with high allele sharing, especially of rare alleles, to have similar effects.

The third part of the penalty in Eq. (3), the MCP [8], encourages overall sparsity in the model while reducing coefficient bias, resulting in most multimarker genotypes that have little apparent effect on the trait to have zero coefficients. The MCP is similar to a thresholding penalty; once our coefficient reaches a certain size, we do not add additional penalization for increasing its size even more. Linear regression struggles with rare covariates: The coefficients can have high variances, and penalization can reduce this variance. Standardization ensures that each covariate is affected more or less equally by the penalization; thus care must be taken when using standardization with penalized regression. We found that standardization led to models that greatly favored rare variables, whereas not standardizing led to models that greatly favored common variables. We choose not to standardize the dummy variables and choose instead to vary the sparsity penalty according to the minor allele frequency. If we expect that rare SNPs have higher relative risk (i.e., they are causal variants) and that we are underpowered to detect these variants otherwise, we can penalize them less heavily than common variants. Note that rare SNPs are already penalized through the group penalty. The concept is similar to that used by Souverein et al. [9], who used a ridge penalty scaled by the haplotype frequency.

### Optimization

The residual sum of squares is a convex function, but our penalty is not quite convex. If our objective function were convex, this would allow us to use the combined local global (CLG) algorithm for optimization [10]. The objective function is minimized using Newton’s algorithm and cyclic coordinate descent [11,12]. Our coefficient update is:(8)

where *n* is the iteration number. When taking the derivative of the penalty function, care must be taken around 0 because the derivative is neither continuous nor differentiable at 0. When our current coefficient  is at 0, we move away from 0 only when certain conditions are met. The allele-sharing penalty has continuous first and second derivatives, but the derivative of the group penalty has a singularity when all coefficients in a group are 0 and the sparsity penalty has a derivative that is discontinuous at 0. We try to move in the direction that improves the objective function, given the other penalty parts; however, this move is not accepted if the derivative of the objective function changes sign (we pass the local minimum). We also do not allow coefficient estimates to take a step that is too large or that changes sign in a single iteration. If our Newton update is:(9)

then let:(10)

where *δ* is chosen by the user.

The sparsity penalty is not convex, because its second derivative is negative when the size of *β* is less than the threshold. The MCP penalty we have designed has a sharp peak when *β* is less than the coefficient threshold and then immediately flattens out. This results in a small, sharp dip in the objective function. We can try to move toward the minimum of the objective function and test whether this point is lower than the peak of the dip. This point is easily found because the derivative of the MCP does not depend on *β* when *β* is over the threshold. If the estimate of the minimum of the objective function is lower than the minimum of the dip, we can move to the new point.

In addition, we run into difficulties with the allele-sharing penalty, which is based on the difference in coefficients, when using cyclic coordinate descent [11,12]. The penalty function is not separable, and thus the descent algorithm may get trapped. For instance, if a change in *β_i_* or *β_j_* cannot improve the objective function, moving them together may. Zhang et al. [13] presented a nice method using Thomas’s algorithm to overcome this problem. However, the group LASSO penalty in our method adds a degree of complexity to the problem, making this model no longer appropriate. Alternatively, we choose to use a fusion cycle similar to the one presented by Friedman et al. [11]; if a coefficient cannot be moved, we look at all other variables in the current group that have effects in the same direction and allele sharing greater than 0 and attempt to move both these coefficients to the smaller of the individual proposed change for the pair.

### Application to Genetic Analysis Workshop 17 data

All association analyses were done on the Genetic Analysis Workshop 17 (GAW17) data with trait Q2, using Age, Smoke, Sex, and reported ethnic group as (unpenalized) covariates over the 200 replicates [14]. Ethnicity was divided into three populations: European (Tuscan and CEPH [European-descended residents of Utah]), Asian (Chinese and Japanese), and African (Luhya and Yoruba). We used the software PLINK [15] to perform single-locus association tests on all 24,487 SNPs in the GAW17 data.

For our method, the 24,487 SNPs were first divided into 3,205 genes. SNPs in long genes were subdivided into smaller windows of approximately 10 SNPs to lower the amount of multimarker genotype diversity within a window, resulting in a total of 4,679 windows. Because most of the alleles were rare, to further limit diversity we chose a dominant genotype coding in which an individual had genotype 0 if he or she had two common alleles and genotype 1 if he or she had one or more rare alleles. This partitioning resulted in 36,612 multimarker genotypes (genetic covariates) to be tested for association. We used the answers to the GAW17 simulation to compute power and false-positive rates. After some exploration of the data, we set *δ* = 0.05, *θ* = (28.0, 7.0, 12.0), and *a* = 0.004. These numbers were chosen so that the overall penalty strength *λ* set near 1 would result in appropriate model sizes for the association analysis and so that groups were not selected preferentially according to their size or the frequency of their elements. Alternatively, we could have opted to make the MCP function constant where the penalty strength varied only with *λ*. In our case, as *λ* increases, the threshold, *aλθ*_3_, increases and the penalty function begins to approach the L1 (LASSO) penalty.

We also compared our method to a version of the sparse group LASSO proposed by Zhou et al. [5],(11)

using the 24,887 SNPs grouped into the 3,205 genes. For this method, we set *λ_E_* = *λ_L_*, as suggested by Zhou et al. [5]. In addition, we applied this method to the multimarker genotypes.

## Results

**Figure 1:**
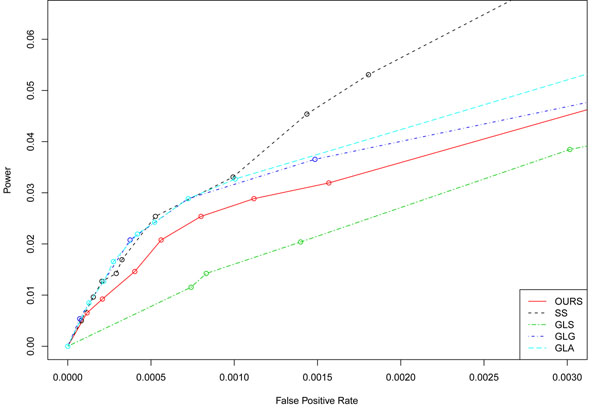
**Power versus false-positive rates for the five methods**. OURS, our method presented in this paper; SS, single-marker test; GLS, sparse group LASSO on single SNPs; GLG, sparse group LASSO on multimarker genotypes; GLA, sparse group LASSO with an allele-sharing penalty on multimarker genotypes.

According to the GAW17 answers, there are 72 causal variants (all of which increase Q2) contained in 13 of the 3,205 genes. Figure 1 is a comparison of the receiver operating characteristic (ROC) curves for the different methods. For the multimarker method, it is somewhat difficult to determine true and false positives. Because we are more interested in gene detection than SNP detection, to compute power, we looked at how well the selected multimarker genotypes (or for the single-locus methods how well the SNPs) tagged a causal gene, that is, a gene containing at least one causal variant. A true detection of a causal gene resulted from any multimarker genotype or SNP contained in the causal gene producing a signal for the given method (nonzero coefficient or below the *p*-value threshold). Any signal from a gene without any causal variants was considered a false positive. Thus there was a maximum of 13 true positives, and 3,205 − 13 = 3,192 true negatives. We considered a variety of penalty strengths for the penalized methods and counted as a signal any genetic variable with a nonzero coefficient. For the single-marker test, we varied the *p*-value threshold and recorded a signal for any SNP with a *p*-value below this threshold.

Figure 1 presents the results for (1) the single-marker analysis, (2) the sparse group LASSO on single SNPs, (3) the sparse group LASSO on multimarker genotypes, (4) our method, and (5) the sparse group LASSO with an allele-sharing penalty where *θ*_2_ = *λ_E_* = *λ_L_*. We see that all methods have low power, with single-marker analysis outperforming the penalized methods and the multimarker methods outperforming the sparse group LASSO on single SNPs. Note that although the differences appear large, we plotted values only for low false-positive rates and low power.

**Figure 2:**
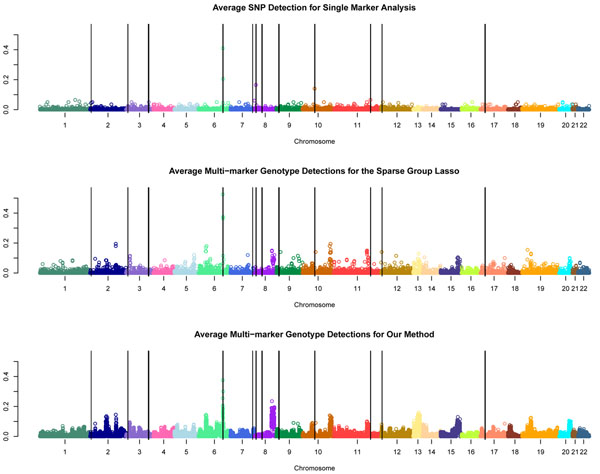
**Detection signals for three different methods**. The three plots present the average detection rates for the single-marker analysis (top), the sparse group LASSO on multimarker genotypes middle), and our method (bottom) for each SNP or multimarker genotype. The *p*-value threshold (5 × 10^−4^) and penalty strengths (16.0 and 0.90, respectively) were chosen to give false-positive rates of about 0.005.

Figure 2 contains plots of the average detection counts for the single-SNP analysis, for the sparse group LASSO on multimarker genotypes, and for our method given a false-positive rate of approximately 0.005 over the replicates. For the single-marker analysis, we counted the average number of replicates in which the *p*-value for the given SNP dropped below the threshold 5 × 10^−4^, the corresponding threshold to give the desired false-positive rate. For the penalized regression methods, the strength of the penalty was *λ_E_* = *λ_L_* = 16.0 for the sparse group LASSO and *λ* = 0.90 for our method. We counted the average number of replicates for which the given multimarker genotype was included in the model.

## Discussion and conclusions

We have presented a novel method for the simultaneous analysis of genes and various individual combinations of alleles within a gene for large data sets. Regression methods allow us to model the effect of several genes simultaneously, and multimarker genotypes are useful not only for capturing untyped variants but also for looking at the effects of combinations of alleles. One advantage of the group penalization approach is that we can encourage variables in a gene to enter the model as a group, because the coefficients within a group are penalized less heavily than they would be if they were in separate groups. With multimarker genotypes (or alternatively haplotypes), the allele-sharing penalty can encourage multimarker genotypes that share rare alleles to have similar coefficients. Thus variants with small effects can be brought into the model, strengthening other variants with small effects.

Unfortunately, our method may not be ideal for the GAW17 data set. Because all the variants have been genotyped, using multimarker genotypes to better tag a causal variant does not necessarily provide an advantage in this situation. In addition, because most of the SNPs are so rare, the analysis might give information similar to single-SNP analysis, yet with a loss of power as a result of the increased number of tests. It is unlikely that someone will have even one minor allele in a small region (many genes having the most common multimarker genotype frequency over 70%), and even less likely that someone will have multiple minor alleles. However, we are pleased that the sparse group LASSO on multimarker genotypes seems to provide some advantage over the sparse group LASSO applied to SNPs within genes and that the method performs similarly to the standard single-marker analysis under strict false-positive rates. We are also happy that the addition of the allele-sharing penalty does not have a detrimental effect (for either our method or the sparse group LASSO). As appealing as the MCP sparsity penalty sounds, in this case it appears to be outperformed by the LASSO (data not shown). One difficulty with our model is the vast number of parameters to be selected by the user. The data must be explored to determine values that will give reasonable results, for example, with null simulations. We would hope that group size and the number of rare variants would not have too much influence over whether or not a group is selected. We think that the parameters we have chosen lead to relatively unbiased results.

A disadvantage of penalized regression methods is that strong sparsity penalties, such as the L1 norm (or LASSO), select only a small subset of a group of highly correlated variables. It could be that the penalized methods are detecting nearby genes that are in linkage disequilibrium with a causal gene instead of the actual causal gene. If we look closely at Figure 2, for the penalized methods there appear to be several signals just adjacent to a causal locus. Thus we may be counting some things as false positive that are in a sense true positives. The single-marker test does not suffer from this artifact because of the individual modeling of the SNPs and thus might have slightly higher power. Although we did not achieve outstanding results, we think that our method is a step in the right direction for modeling effects for rare variants. We hope that increased sample size and better sequencing technologies will make this and similar methods viable options in the future.

## Competing interests

The authors declare that there is/are no competing interest(s).

## Authors’ contributions

KLA developed and programmed the methods, performed the analysis, and drafted the manuscript. CM performed the analysis in PLINK. HJC gave advice on methodology and analysis, and assisted in editing the manuscript.
